# Common Variable Immunodeficiency Revealed by Bronchiectasis: A Case Report

**DOI:** 10.7759/cureus.81647

**Published:** 2025-04-03

**Authors:** Mohamed Lakhal, Touria El Baroudi, Meriem Rhazari, Sara Gartini, Afaf Thouil, Hatim Kouismi

**Affiliations:** 1 Department of Pulmonology, Research and Medical Sciences Laboratory, Centre Hospitalier Universitaire (CHU) Mohammed VI, Faculty of Medicine and Pharmacy, Mohammed I University, Oujda, MAR

**Keywords:** bacterial infections, bronchiectasis, common variable immunodeficiency, immunoglobulin, lymphoproliferation

## Abstract

We report a case of a 38-year-old female with a past medical history of lymph node tuberculosis treated for six months in 2015, history of bronchiectasis from the past six years, and recurrent sinopulmonary infections, who was subsequently diagnosed to have common variable immunodeficiency (CVID). She had reduced levels of immunoglobulins during our diagnostic workup, after ruling out hematological malignancy and solid tumors. CVID is a highly heterogeneous group of disorders characterized by a primary defect in immunoglobulin production and an inability to mount a specific humoral response against exogenous antigens. The most frequently reported pulmonary manifestations of CVID are infectious pneumonias. Bronchiectasis, resulting from recurrent infections, is the third most common pulmonary manifestation observed in CVID patients, following pneumonia and bacterial bronchitis. The therapeutic management of CVID focuses on evaluating complications, with particular emphasis on the risks of bronchial dystrophy (bronchiectasis) and bronchial colonization by antibiotic-resistant pathogens. Respiratory physiotherapy is a key element in the management of bronchial suppuration. Treatment for CVID mainly consists of immunoglobulin replacement therapy, administered intravenously or subcutaneously, which must be given for life.

## Introduction

Common variable immunodeficiency (CVID) forms a heterogeneous group of diseases characterized by a primary defect in immunoglobulin production and an inability to develop a specific humoral response against exogenous antigens [1]. According to the WHO, CVID is defined as the association of hypogammaglobulinemia, a decreased ability to produce antibodies after antigenic stimulation, and an increased frequency of infectious events, with a normal B lymphocyte count [2]. The most common and well-known pulmonary manifestations of CVID are infectious pneumopathies, reported in 50 to 75% of patients in CVID cohort studies [3]. Bronchiectasis (BE), resulting from repeated infections, is the third most common pulmonary manifestation observed in patients with CVID, after pneumonia and bacterial bronchitis [3]. We report a case of a young patient with a chronic history of bronchiectasis, who was subsequently diagnosed with CVID.

## Case presentation

This is a case of a 38-year-old patient with a history of lymph node tuberculosis (diagnosed as giant cell granuloma after adenectomy) diagnosed in 2015, treated for six months and declared cured. She has been followed for bronchiectasis (BE) and has been on inhaled treatment for six years, with several infectious exacerbations requiring hospitalization. The patient was admitted to our department for an etiological diagnosis of her BE.

Clinically, the patient was in good general condition with a performance score of 1 and stable respiratory status, with an oxygen saturation of 95% on room air. The pulmonary examination revealed the presence of bilateral bronchial rales. The examination of the lymphatic areas showed multiple supraclavicular lymphadenopathies, the largest measuring 14 x 13 mm on the left side. The rest of the clinical examination was unremarkable, particularly regarding the skin and digestive systems.

**Figure 1:**
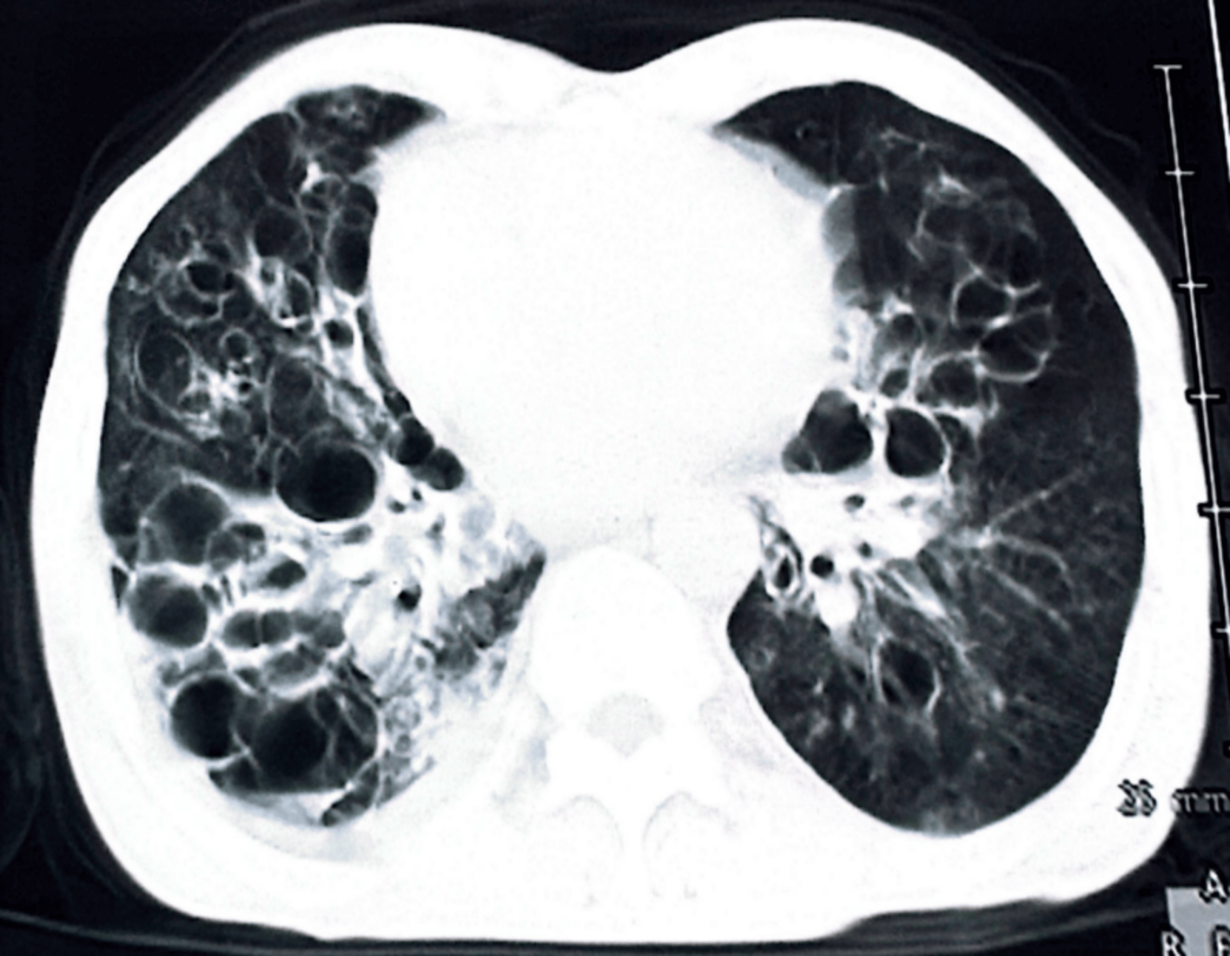
A scannographic image with a parenchymal window showing bilateral diffuse cystic bronchiectasis foci, associated with a condensation focus in the right lower lobe.

A chest CT scan was performed initially, revealing the presence of diffuse bilateral cystic BE foci, associated with a consolidation focus in the right lower lobe (Figure 1), as well as multiple mediastinal lymphadenopathies, the largest measuring 30 x 13 mm in the right laterotracheal area.

As part of the etiological workup, a complete blood count, tests for antinuclear antibodies (ANAs), circulating antibodies, anticardiolipin antibodies, as well as viral serology for hepatitis B, hepatitis C, and HIV were performed. The 24-hour proteinuria was normal. The protein electrophoresis (PEP) showed hypogammaglobulinemia. The immunoglobulin subclass testing revealed a decrease in IgG, IgA, and IgM levels. The albumin level was normal. A bone biopsy (BOM) was performed, which was suggestive of a reactive marrow.

**Figure 2:**
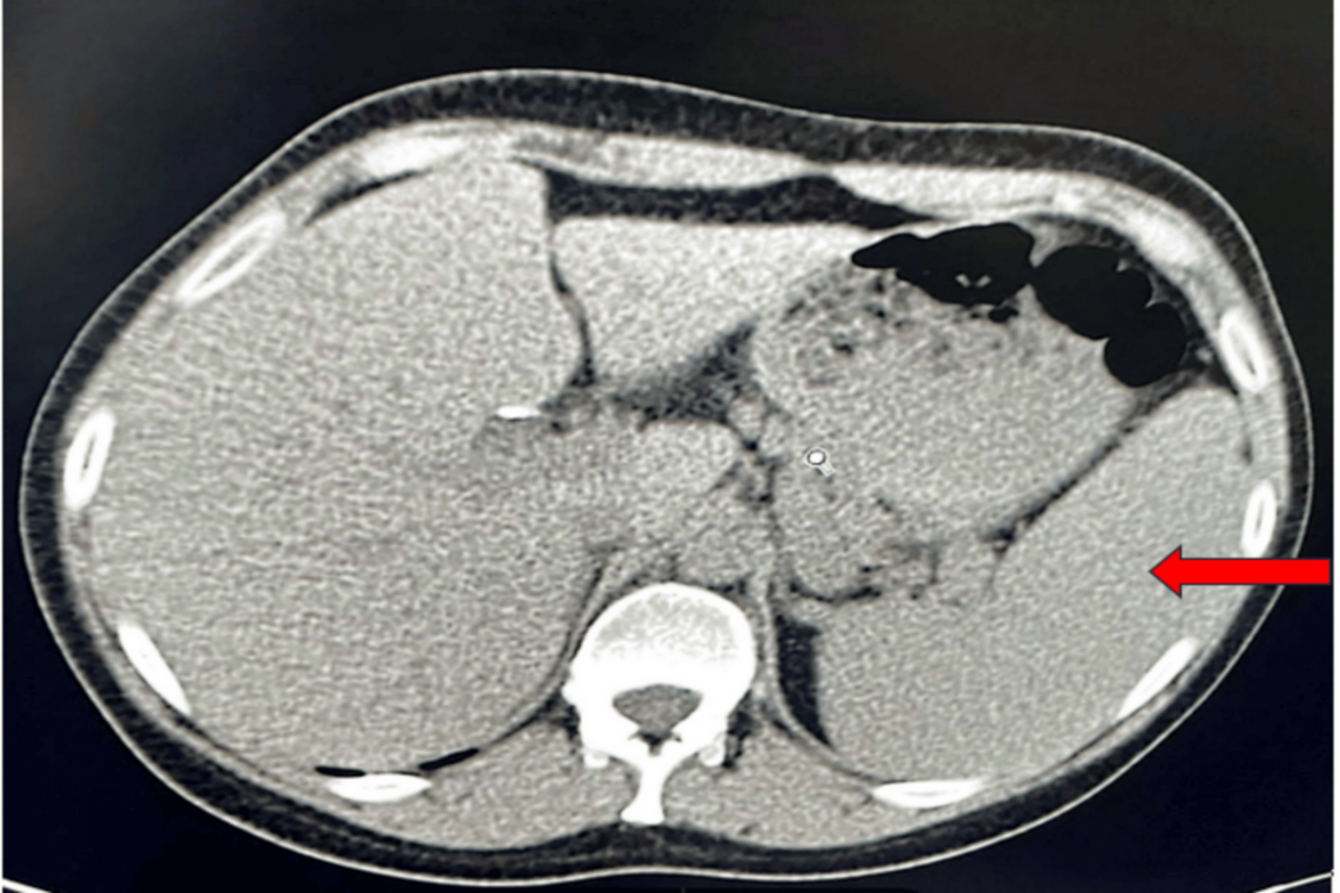
Abdominal CT scan showing splenomegaly. Red arrow: Splenomegaly measuring 115 mm in size with homogeneous density.

In the context of investigating the cause of hypogammaglobulinemia, a complementary CT scan of the cervico-thoraco-abdomino-pelvic region was performed. This revealed the presence of multiple supraclavicular, subpectoral, and bilateral axillary lymphadenopathies. A splenomegaly of 115 mm in size with homogeneous density was also noted (Figure 2).

**Figure 3:**
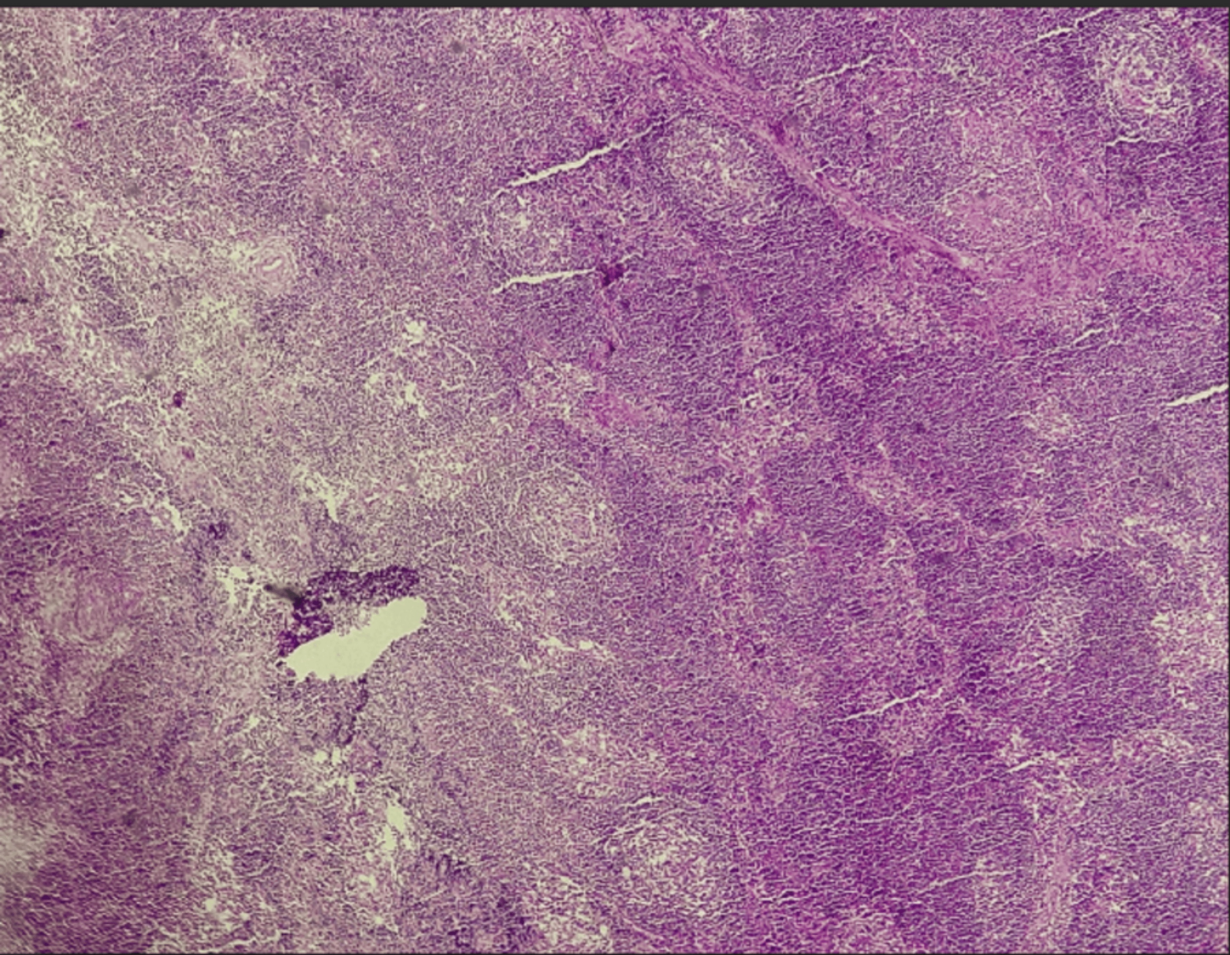
Histological image of lymph node parenchyma showing lymphoid follicular hyperplasia (hematoxylin & eosin, x4).

Flexible bronchoscopy to search for acid-fast bacilli (AFB) and neoplastic cells revealed no abnormalities. A mediastinoscopy was performed to biopsy the mediastinal lymphadenopathies to rule out a lymphomatous cause or metastasis from a solid neoplasm. The results showed lymphoid follicular hyperplasia without visible granulomas (Figure 3).

The diagnosis of CVID was made based on hypogammaglobulinemia, increased susceptibility to infections (+++), and a decrease in immunoglobulin subclasses, after excluding secondary causes of hypogammaglobulinemia, particularly hematologic diseases and solid tumors. The patient was referred to the internal medicine department for further management.

## Discussion

CVID refers to a heterogeneous group of immunodeficiencies whose mechanisms are not yet fully understood. Its clinical manifestations and immunological characteristics vary greatly from one patient to another [4]. Most often, the hypogammaglobulinemia characteristic of this disease leads to recurrent bacterial infections due to a defect in antibody production. It is defined by a serum IgG level lower than 5 g/dL, associated with a deficiency in IgA and/or IgM, and most often, by an absence of response to vaccine antigens. It is essential to rule out other causes of hypogammaglobulinemia, such as primary immunodeficiencies.

The prevalence of CVID varies across studies, ranging from one in 50,000 to one in 100,000 inhabitants [5]. It affects both men and women equally. The diagnosis can be made at any age, but the peak frequency occurs between the ages of 20 and 30 years. The first infectious episodes often occur earlier in childhood or adolescence [5].

The presence of at least one of the following elements is also considered a potential indicator of CVID: accumulated susceptibility to infections, autoimmune manifestations, granulomatous disease, unexplained polyclonal lymphoproliferation, a family member with an antibody deficiency, and a marked decrease in IgG and IgA, with or without a decrease in IgM. Additionally, the presence of at least one of the following should be observed: poor vaccine response, decreased memory B cells (<70% of normal values for age), and exclusion of secondary causes of hypogammaglobulinemia [6].

The clinical and biological manifestations that allow for the diagnosis of this disease are extremely varied, which gives it a heterogeneous nature. Respiratory manifestations, which are the most common signs in CVID, are of an infectious nature and present as recurrent bacterial pneumonias and bronchial suppurations. After a few years, this can lead to BE. The most frequently responsible pathogens are encapsulated bacteria, such as *Haemophilus influenzae*, *Streptococcus pneumoniae*, and *Moraxella catarrhalis* [3].

BE is among the most frequent complications and is partly the result of iterative infections of the pulmonary parenchyma [7]. These BEs are most often bilateral and diffuse, typically cystic in nature, without apical or basal predominance [8]. Their risk is increased by the delayed diagnosis of the underlying immunodeficiency. They are more common in cases of complete IgA deficiency, low IgM levels, or a profound deficiency in memory B lymphocytes [9]. Functionally, they frequently present with an obstructive ventilatory disorder. The progression of these BEs may be marked by colonization and superinfections with pathogens such as *Staphylococcus aureus* and *Pseudomonas aeruginosa* [3].

Pulmonary granulomatosis and lymphoid infiltrations are rarer but can sometimes be revealing. Pulmonary CT imaging shows various patterns, such as reticulations, nodules, lymphadenopathy, ground-glass opacities, or fibrosis, which may persist or worsen despite immunoglobulin replacement therapy [3].

A lymphoproliferative syndrome is found in 20% of patients with CVID. It involves enlargement of lymphoid organs, such as lymph nodes or the spleen, but sometimes also the digestive tract or pulmonary parenchyma, in the form of follicular hyperplasia, as seen in our patient [10].

The therapeutic management of CVID focuses on evaluating complications, particularly emphasizing the risks of bronchial dystrophy (BE) and bronchial colonization by antibiotic-resistant pathogens. Respiratory physiotherapy is a key element in managing bronchial suppuration [11].

The treatment of CVID primarily relies on antibody replacement therapy, through IV or subcutaneous immunoglobulin infusion, which must be administered for life. This treatment helps restore immune defense and regulation mechanisms, as well as prevent or treat the main infectious and autoimmune complications [12]. Replacement therapy is initiated immediately in the following cases: if the IgG level is below 2 g/L, in the presence of a history of severe infections (such as meningitis, sepsis, and pneumonia), and in the presence of certain non-infectious complications such as enteropathy, granulomatous-lymphocytic interstitial lung disease (GLILD), BE, or autoimmune cytopenias [12].

## Conclusions

Pulmonologists should perform serum protein electrophoresis, not only in the case of classic infectious complications of CVID (recurrent pneumococcal pneumonia, bronchiectasis), but also in cases of diffuse interstitial pneumonia and pulmonary lymphoma. Early detection is essential to reduce the morbidity and mortality associated with CVID.
